# Quantitative Pharmacokinetic Analysis of Dynamic Contrast-Enhanced Perfusion in Multiparametric Prostate MRI: An Exploratory Intra-Patient Study Differentiating Malignant Lesions from Normal Tissue

**DOI:** 10.3390/diagnostics16193075

**Published:** 2026-09-22

**Authors:** Muhammed Sonkaya, İlyas Dündar

**Affiliations:** Department of Radiology, Faculty of Medicine, Van Yüzüncü Yıl University, 65040 Van, Türkiye; muhammedsonkaya@hotmail.com

**Keywords:** prostate cancer, multiparametric MRI, dynamic contrast enhancement, pharmacokinetic analysis, PI-RADS v2.1, Gleason score

## Abstract

**Background/Objectives**: To quantitatively evaluate pharmacokinetic parameters derived from dynamic contrast-enhanced magnetic resonance imaging (DCE-MRI) in differentiating prostate malignant lesions from normal appearing prostatic tissue, and to analyze their correlation with Gleason scores (GS). **Methods**: A retrospective analysis was performed on 51 patients with initial biopsy-confirmed prostate cancer who underwent multiparametric MRI (mpMRI) between May 2021 and April 2024 for elevated serum prostate-specific antigen (PSA) levels and/or suspicious digital rectal examination (DRE) findings. Forty-three eligible patients with histopathologically confirmed prostate adenocarcinoma (GS ≥ 6) who met all strict imaging criteria were included. Demographic parameters, serum PSA, prostate volume, PSA density (manual: PSA-D*_m_*, workstation: PSA-D*_w_*), and lesion dimensions were recorded. Free-hand 2D regions of interest (ROIs) were placed on index lesions and normal peripheral zone tissue on a dedicated workstation (1.5-T MRI, syngo.via Tofts model) to compute quantitative and semi-quantitative pharmacokinetic parameters (*K*^trans^, *K_ep_*, *iAUC*, *V*_e_, *χ*^2^). Statistical evaluations utilized non-parametric tests, Spearman’s rank correlation, and clustered ROC analysis with patient-level bootstrapping. **Results**: Mean patient age was 64.21 ± 8.82 years. Significant differences were identified across GS groups regarding serum PSA, PSA-D*_m_*, and PSA-D_w_ (*p* < 0.05). Group 3 (GS ≥ 8) demonstrated significantly higher median PSA (12.99 ng/mL) compared to Groups 1 and 2 (*p* = 0.004). For predicting high-risk disease (GS ≥ 8), ROC analysis for PSA (cut-off 7.25 ng/mL) yielded an area under the curve (AUC) of 0.798, whereas *K*^trans^ performed poorly (AUC = 0.560). Conversely, for intra-patient paired comparisons distinguishing malignant lesions from normal background parenchyma, all quantitative DCE-MRI parameters (*K*^trans^, *K_ep_*, *iAUC*, *V*_e_, *χ*^2^) were significantly higher in malignant lesions (all *p* < 0.001, surviving stringent Bonferroni correction). Among pharmacokinetic parameters, *K*^trans^ (cut-off: 0.105, AUC = 0.853, sensitivity: 81.4%, specificity: 76.7%) and *χ*^2^ (cut-off: 0.049, AUC = 0.844, sensitivity: 79.1%, specificity: 76.7%) exhibited the highest discriminative performance for identifying malignancy. Crucially, however, DCE-MRI parameters demonstrated no statistically significant correlation with Gleason scores (all *p* > 0.05) and failed to independently predict high-grade disease. **Conclusions**: Quantitative DCE-MRI pharmacokinetic parameters effectively differentiate malignant prostate lesions from normal parenchyma within this specific cohort, but they do not correlate with histological grade. Furthermore, the derived diagnostic cut-offs are highly protocol-dependent, reflecting 1.5-T hardware and 2D ROI sampling. Robust cross-platform standardization and prospective 3D whole-gland validations are strictly required before these functional metrics can be reliably incorporated into clinical workflows.

## 1. Introduction

Prostate cancer (PCa) represents the most common non-dermatologic malignancy diagnosed in men worldwide and stands as the fifth leading cause of cancer-related mortality [1]. Aside from skin cancers, prostate cancer (PCa) ranks as the most common malignancy and the second leading cause of cancer-related death among men in the United States [2]. Its growing incidence and mortality rates over the last twenty years highlight it as a major global health concern [3]. Although early detection is crucial, traditional screening approaches like the digital rectal examination (DRE) and prostate-specific antigen (PSA) tests suffer from low specificity. Non-cancerous conditions such as prostatitis and Benign Prostatic Hyperplasia can also increase PSA levels, frequently resulting in unwarranted biopsies [4,5,6].

The primary determinants of prognosis in PCa are the pathological Gleason score (GS) and clinical stage at initial presentation. Organ-confined PCa responds favorably to definitive local therapy; however, delayed diagnosis permitting extraprostatic extension or systemic dissemination significantly increases disease-specific morbidity and mortality [3]. The integration of multiparametric magnetic resonance imaging (mpMRI) into clinical practice has revolutionized PCa screening and diagnosis, facilitating early detection of aggressive disease and reducing cancer-specific mortality [3].

Currently, mpMRI is universally recognized as the optimal non-invasive modality for prostate imaging. It provides high-resolution visualization of prostatic zonal architecture, anatomical localization of suspicious lesions, assessment of extraprostatic extension, and evaluation of pelvic lymph nodes [7]. Although the Prostate Imaging Reporting and Data System (PI-RADS) v2.1 has standardized reporting guidelines [8], and higher scores (PI-RADS 4 and 5) generally demonstrate high accuracy for detecting clinically significant disease [9], evaluation remains inherently qualitative and observer-dependent, leading to diagnostic overlap between benign mimics and malignant lesions [7].

Tumor angiogenesis, characterized by abnormal vascular proliferation and heightened capillary permeability, is a hallmark of PCa progression. Dynamic Contrast-Enhanced MRI (DCE-MRI), an essential functional component of the mpMRI protocol, offers valuable quantitative and semi-quantitative metrics of tissue microvascular perfusion and microvascular wall permeability [10]. Dynamic contrast-enhanced MRI (DCE-MRI) quantifies the transit of low-molecular-weight gadolinium chelates between intravascular plasma space and the extravascular extracellular space (EES) through rapid serial T1-weighted acquisitions [11,12]. The physiological processes governing dynamic contrast kinetics include tissue perfusion, microvascular blood flow, endothelial permeability, and EES volume fraction. Standard quantitative parameters derived from Tofts pharmacokinetic modeling comprise the volume transfer constant (*K*^trans^), which reflects endothelial permeability and blood flow; the EES volume fraction (*V_e_*); and the rate constant (*K_ep_* = *K*^trans^/*V_e_*), describing back-flux from EES to blood plasma [10,13]. The semi-quantitative parameter *iAUC* represents total contrast accumulation during the initial 60 s post-injection [10]. In malignant prostate tissue, neo-angiogenesis increases capillary density, vessel tortuosity, and endothelial pore size, while simultaneously reducing the EES space due to dense cellular packing. Consequently, malignant foci characteristically display elevated *K*^trans^ and *K_ep_* values compared to normal parenchymal tissue [11,14].

The primary objective of this retrospective study is to quantitatively assess the diagnostic performance of pharmacokinetic parameters derived from DCE-MRI in differentiating histopathologically confirmed malignant prostate lesions from normal-appearing prostatic tissue in a paired intra-patient design. Based on previous literature suggesting a relationship between tumor microvascularity and clinical aggressiveness, a secondary objective was to investigate whether these quantitative parameters (*K*^trans^, *K_ep_*, *iAUC*, *V*_e_, *χ*^2^) correlate with pathological Gleason scores and possess the capability to independently differentiate high-grade (GS ≥ 8) from low-to-intermediate risk (GS 6–7) disease.

## 2. Materials and Methods

### 2.1. Ethical Approval

Ethical approval for this retrospective study was granted by the Non-Interventional Clinical Research Ethics Committee of Van Yüzüncü Yıl University (Decision Date: 10 May 2024; Protocol Code: 2024/05-22). All procedures were conducted in strict adherence to the ethical principles outlined in the Declaration of Helsinki.

### 2.2. Patient Selection and Study Cohort

A retrospective database search was initially performed to identify patients who underwent multiparametric prostate MRI (mpMRI) at a tertiary university hospital between May 2021 and April 2024 due to elevated serum PSA levels (>4.0 ng/mL) and/or suspicious DRE findings. This initial screening assessed a total of 813 consecutive male patients for eligibility.

The primary inclusion criteria for the study required: (1) a completed mpMRI protocol adhering to PI-RADS v2.1 specifications; (2) a subsequent TRUS-guided cognitive MRI biopsy and/or radical prostatectomy within 3 months of the imaging (specifically, pathological grading was based on TRUS-guided biopsy in 32 patients and radical prostatectomy in 11 patients); and (3) a histopathologically confirmed diagnosis of prostate adenocarcinoma with a Gleason score of 6 or higher (GS ≥ 6) derived from these specimens. Based on these criteria, a total of 762 patients were excluded from the initial cohort because they either did not undergo a subsequent biopsy or were diagnosed with benign conditions/low-grade disease (Gleason score < 6).

This initial exclusion step established a core group of 51 patients with biopsy-confirmed prostate cancer (GS ≥ 6). A secondary exclusion phase was then applied to ensure the feasibility and reliability of the quantitative image analysis. Eight patients were subsequently excluded from this core group for the following specific reasons: an extreme outlier serum PSA value measured at >100,000 ng/mL (*n* = 1), unmeasured or unretrievable baseline serum PSA data (*n* = 1), the absence of a significant or distinct focal target lesion on the mpMRI evaluation (*n* = 2), and technical reasons (such as imaging artifacts) that impeded successful pharmacokinetic modeling on the post-processing workstation (*n* = 4).

Following this rigorous selection process, a final analytical cohort comprising 43 eligible patients, aged 47 to 85 years, was successfully established (Figure 1).

### 2.3. Prostate mpMRI Acquisition Protocol

Prior to imaging, patients received comprehensive procedural instructions. History of adverse reactions to gadolinium-based contrast agents was evaluated. Patients were instructed to observe sexual abstinence for at least 72 h prior to the exam to ensure adequate seminal vesicle distension. Bowel preparation with a mild laxative/enema and pre-scan voiding were recommended to minimize susceptibility artifacts caused by rectal gas/feces and bladder motion.

All examinations were performed on a 1.5-Tesla MRI system (Magnetom Altea, Siemens Healthineers, Erlangen, Germany) equipped with an 18-channel pelvic phased-array body coil. The imaging protocol fully complied with PI-RADS v2.1 standards, comprising: multiplanar T2-weighted turbo spin-echo (T2WI) sequences in axial, coronal, and sagittal planes; axial diffusion-weighted imaging (DWI) using b-values of 50, 400, 800, and 1600 s/mm^2^ with automated apparent diffusion coefficient (ADC) map generation; axial non-fat-saturated T1-weighted imaging (T1WI); and dynamic contrast-enhanced (DCE) sequences. Post-contrast axial T1WI encompassing pelvic lymph node chains was acquired for local staging (Table 1).

For the DCE acquisition, all patients uniformly received a standard dose of 0.1 mmol/kg of a macrocyclic gadolinium-based contrast agent (either gadobutrol [Gadovist, Bayer Healthcare, Leverkusen, Germany] or gadoteric acid [Clariscan, Opakim, Istanbul, Türkiye]). The contrast agent was administered intravenously via a power injector at a fixed rate of 3.0 mL/s, followed by a 20 mL saline flush. Sequential dynamic axial images (slice thickness 3 mm, temporal resolution 7 s) were acquired over a total scanning duration of 240–300 s.

### 2.4. Image Analysis and Morphometric Measurements

All prostate mpMRI datasets were independently and consensus-reviewed on a dedicated workstation (SYNGO.VIA, VB40 Software, Siemens Healthineers, Forcheim, Germany) by two abdominal radiologists with 5 and 11 years of experience in prostate imaging, blinded to final histopathology but unblinded to age and serum PSA levels.

Prostate volume was determined manually using the standard prolate ellipsoid formula:

Prostate Volume (PV*_m_*) = Anteroposterior Length × Transverse Length × Craniocaudal Length × 0.52

Manual PSA density (PSA-D*_m_*) was calculated as serum PSA divided by PV*_m_*. In addition, datasets were processed in SYNGO.VIA by placing sequential free-hand regions of interest (ROIs) encompassing the entire prostate gland from apex to base across contiguous axial slices. The software automatically generated dynamic workstation prostate volume (PV*_w_*) and workstation PSA density (PSA-D*_w_*} = PSA/PSA-V_w_).

Target index lesions were scored per PI-RADS v2.1 criteria: 2 patients were classified as PI-RADS 2, 2 as PI-RADS 3, 15 as PI-RADS 4, and 24 as PI-RADS 5. Free-hand ROIs were delineated around the perimeter of the index lesion across multiple axial slices to determine automated 3D lesion volume.

### 2.5. Pharmacokinetic Parameter Analysis

Pharmacokinetic analysis was conducted using the standard Tofts pharmacokinetic model implemented on the SYNGO.VIA post-processing software. To ensure reproducibility and eliminate operator-dependent variability, a population-based Arterial Input Function (AIF) (Parker model) integrated within the Siemens syngo.via post-processing workstation was utilized for all quantitative parameter derivations. A free-hand ROI was drawn over the maximum cross-sectional area of the index lesion on dynamic axial sequences. Quantitative parameters—volume transfer constant (*K*^trans^, min^−1^), extravascular extracellular space (EES) volume fraction (*V_e_*), and rate constant (*K_ep_* = *K*^trans^/*V_e_*, min^−1^)—along with semi-quantitative metrics including initial area under the concentration curve in the first 60 s (*iAUC*) and goodness-of-fit error (*χ*^2^) were extracted (Figure 2).

For internal comparison, one or two free-hand ROIs were placed over morphologically normal, homogeneous hyperintense peripheral zone tissue on T2WI in the same slice/zone (Figure 2). When two control ROIs were drawn, mean values were calculated. Pharmacokinetic metrics recorded for lesions were designated with the prefix “L-” (e.g., L-*K*^trans^), whereas non-tumoral reference tissues were prefixed with “N-” (e.g., N- *K*^trans^).

Interobserver agreement for ROI delineation and pharmacokinetic parameter extraction was evaluated using the Intraclass Correlation Coefficient (ICC). The reproducibility between the two evaluating radiologists demonstrated excellent agreement, with an overall ICC of 0.88 (95% CI: 0.82–0.93).

### 2.6. Patient Subgrouping

To evaluate pharmacokinetic variation across tumor grade categories, the 43 patients were stratified into three histological groups based on biopsy/surgical Gleason score (GS):

Group 1: GS = 6 (ISUP Grade Group 1; *n* = 20).

Group 2: GS = 7 (ISUP Grade Groups 2–3; *n* = 9).

Group 3: GS ≥ 8 (ISUP Grade Groups 4–5; *n* = 14).

Additionally, patients were dichotomized into Group A (GS 6–7; low-to-intermediate risk, *n* = 29) versus Group B (GS ≥ 8; high risk, *n* = 14) for receiver operating characteristic (ROC) comparative analysis. This dichotomization into low-to-intermediate risk (GS 6–7) and high-risk (GS ≥ 8) disease was based on established clinical risk stratification guidelines, which identify GS ≥ 8 as a critical threshold for significantly increased risk of extraprostatic extension, metastasis, and prostate cancer-specific mortality [15].

### 2.7. Statistical Analysis

Statistical evaluations were executed using IBM SPSS Statistics for Windows, Version 24.0 (IBM Corp., Armonk, NY, USA). The normality of continuous variables was assessed using the Shapiro–Wilk test. Normally distributed variables were reported as Mean ± Standard Deviation (SD), Minimum, and Maximum; whereas non-normally distributed continuous variables were presented as Median and Interquartile Range (IQR). Categorical variables were summarized as counts and percentages. Due to the non-normal distribution of several variables and the small sample sizes in subgroups, the non-parametric Kruskal–Wallis test was employed for multi-group comparisons (Groups 1, 2, and 3). The Wilcoxon signed-rank test was utilized to assess paired differences between lesion (“L”) and normal tissue (“N”) parameters within patient cohorts. Bivariate correlations were examined using Spearman’s rank correlation coefficient. Diagnostic performance and optimal cut-off points were derived from Receiver Operating Characteristic (ROC) curve analysis using the Youden Index. Furthermore, to appropriately account for the intra-patient correlation of paired lesion and normal tissue measurements, a patient-level bootstrap technique with 1000 iterations was applied to the ROC analysis to derive stable confidence intervals. A two-tailed *p*-value < 0.05 was defined as statistically significant; however, to adjust for the multiple comparisons of the five pharmacokinetic parameters between paired tissues, a Bonferroni correction was applied, establishing a strict significance threshold of *p* < 0.01 for these specific analyses.

## 3. Results

Demographic characteristics and baseline volumetric/PSA measurements for all 43 patients are detailed in Table 2. The mean patient age was 64.21 ± 8.82 years (range: 47–85 years). Following normality testing, non-normally distributed continuous variables are reported as median and interquartile range (IQR). The median overall serum PSA level was 6.55 ng/mL (IQR: 5.46–10.97 ng/mL). Median manual prostate volume (PV*_m_*) and workstation prostate volume (PV*_w_*) were 58.00 mL (IQR: 44.95–79.25 mL) and 57.81 mL (IQR: 39.05–73.58 mL), respectively. Corresponding median PSA densities were 0.13 (IQR: 0.07–0.26) for PSA-Dm and 0.14 (IQR: 0.08–0.23) for PSA-D*_w_*.

Comparative analysis among Gleason score groups (Group 1, Group 2, and Group 3) revealed no statistically significant differences regarding age, prostate volume (PV*_m_* or PV*_w_*), or lesion volume (*p* > 0.05; Table 3). However, serum PSA levels and both PSA density calculations (PD*_m_* and PD*_w_*) differed significantly across groups (*p* = 0.004, *p* = 0.019, and *p* = 0.034, respectively). Post-hoc pairwise testing demonstrated that Group 3 (GS ≥ 8) had markedly higher median PSA (12.99 ng/mL), PSA-D*_m_* (0.34), and PSA-D*_w_* (0.31) compared to Group 1 (GS 6) and Group 2 (GS 7) (*p* < 0.05 for all pairwise comparisons against Group 3; Table 3).

To assess the utility of clinical parameters in discriminating high-grade prostate cancer (Group B: GS ≥ 8) from low-to-intermediate grade disease (Group A: GS 6–7), ROC curves were constructed (Table 4). Serum PSA demonstrated the highest diagnostic accuracy with an AUC of 0.798 (*p* = 0.002). At a cut-off value of 7.25 ng/mL, serum PSA achieved a sensitivity of 71.4% and specificity of 69.0%. Manual PSA density (PSA-D*_m_*) yielded an AUC of 0.765 (cut-off: 0.135, sensitivity: 71.4%, specificity: 62.1%, *p* = 0.005), while workstation PSA density (PSA-D*_w_*) showed an AUC of 0.739 (cut-off: 0.145, sensitivity: 64.3%, specificity: 58.6%, *p* = 0.012).

In the ROC analysis distinguishing high-risk (GS ≥ 8) from low-to-intermediate risk (GS 6–7) disease, clinical parameters outperformed DCE-MRI metrics. While serum PSA achieved the highest diagnostic accuracy (AUC = 0.798), pharmacokinetic parameters demonstrated poor discriminative performance (AUC ranging from 0.516 for *K_ep_* to 0.634 for *iAUC*) and failed to reach statistical significance (all *p* > 0.05). This indicates that while DCE-MRI parameters are excellent for differentiating malignant from normal tissue, they may not reliably predict high-grade pathology independently within this limited cohort size.

Quantitative and semi-quantitative pharmacokinetic parameters measured within target lesions (L-) versus healthy background tissues (N-) across all groups are detailed in Table 5. Due to the non-normal distribution of several variables, Wilcoxon signed-rank tests were utilized for intra-patient paired comparisons. This analysis demonstrated that lesion values for L- *K*^trans^, L-*K_ep_*, L-*V_e_*, L-*iAUC*, and L-*χ*^2^ were statistically significantly higher than corresponding non-tumoral parenchymal values (N-) across the overall patient population and within almost all individual Gleason score subgroups. Notably, the differences across the overall cohort were highly significant (all *p* < 0.001), demonstrating robust statistical significance that remained perfectly valid even after applying the stringent Bonferroni correction for multiple comparisons (adjusted threshold *p* < 0.01) (Table 5).

The diagnostic performance of individual pharmacokinetic parameters in distinguishing malignant lesions from normal tissue was evaluated using a clustered ROC analysis with a patient-level bootstrap approach to appropriately account for the intra-patient paired nature of the measurements (Table 6). All evaluated DCE parameters demonstrated statistically significant diagnostic capability (*p* < 0.001). The volume transfer constant (*K*^trans^) exhibited the highest overall performance with an AUC of 0.853. At a cut-off threshold of 0.105 min^−1^, *K*^trans^ yielded 81.4% sensitivity and 76.7% specificity. Goodness-of-fit (*χ*^2^) ranked second with an AUC of 0.844 (cut-off: 0.049, sensitivity: 79.1%, specificity: 76.7%), followed by *iAUC* (AUC: 0.808, cut-off: 0.136, sensitivity: 72.1%, specificity: 69.8%), and *K_ep_* (AUC: 0.754, cut-off: 0.405 min^−1^, sensitivity: 69.8%, specificity: 67.4%). Extravascular extracellular space fraction (*V_e_*) displayed moderate performance with an AUC of 0.706 (cut-off: 0.227, sensitivity: 76.7%, specificity: 65.1%).

A Spearman rank correlation analysis revealed no statistically significant correlation between the quantitative DCE-MRI parameters (*K*^trans^, *K_ep_*, *V_e_*, *iAUC*, *χ*^2^) and the Gleason score groups (all *p* > 0.05). Similarly, Kruskal–Wallis testing demonstrated no significant differences in perfusion metrics across GS 6, GS 7, and GS ≥ 8 subgroups, likely due to the small sample size (Table 7).

## 4. Discussion

In this exploratory intra-patient comparative study, we demonstrated that all quantitative and semi-quantitative DCE-MRI parameters *K*^trans^, *K_ep_*, *V_e_*, *iAUC*, and *χ*^2^) were significantly higher in MRI-visible malignant prostate lesions compared to normal appearing background tissue (all *p* < 0.001, maintaining high statistical significance even after Bonferroni correction). Among these parameters, *K*^trans^ achieved the highest discriminative performance in our clustered ROC analysis (AUC = 0.853; sensitivity: 81.4%; specificity: 76.7% at a cut-off of 0.105 min^−1^), closely followed by the goodness-of-fit metric *χ*^2^ (AUC = 0.844; cut-off: 0.049; sensitivity: 79.1%; specificity: 76.7%). However, because our cohort consisted exclusively of patients with pathologically confirmed cancer and utilized imaging-defined normal tissue as an internal control, these AUCs and cut-off values represent relative intra-patient microvascular differences rather than absolute clinical diagnostic efficacies for generalized prostate cancer screening.

These findings align with several reports in the literature. Beyhan et al. [16] evaluated 3T mpMRI perfusion parameters in prostate lesions and reported significantly elevated mean *K*^trans^, *K_ep_*, *V_e_*, *iAUC*, and *χ*^2^ values in peripheral zone malignant lesions relative to healthy tissue (*p* = 0.00, *p* = 0.02, *p* = 0.00, and *p* = 0.02, respectively), without significant differences for *V_e_* (*p* = 0.12). Cho et al. [17] similarly confirmed that malignant tumors exhibit significantly higher *K*^trans^, *K_ep_*, and *iAUC* values.

Interestingly, while classic tumor biology dictates that dense cellular packing should reduce the extravascular extracellular space (EES), our study—along with Riches et al. [18] and Beyhan et al. [16]—observed paradoxically elevated *V_e_* values in malignant lesions. This apparent inconsistency can be explained by the biological heterogeneity of prostate cancer; the destruction of normal glandular architecture, the presence of microscopic focal necrosis, and the accumulation of luminal secretions in certain architectural patterns can effectively expand the interstitial space [19]. Additionally, variations in temporal resolution or the mathematical assumptions of utilizing a population-based AIF can influence *V_e_* estimation.

Furthermore, the goodness-of-fit metric (*χ*^2^) demonstrated an unexpectedly high discriminative ability (AUC = 0.844). Clinically, *χ*^2^ quantifies the deviation of the actual tissue contrast enhancement curve from the idealized Tofts compartmental model. We hypothesize that the highly chaotic, heterogeneous, and tortuous neo-angiogenic microvasculature typical of malignant lesions exhibits complex flow dynamics (such as arterio-venous shunting and extreme permeability). As suggested by established pharmacokinetic modeling principles [5,10,11], these complex dynamics deviate significantly from standard kinetic modeling, resulting in larger residual errors (higher *χ*^2^) compared to the orderly vascular bed of normal tissue. Thus, elevated *χ*^2^ may serve as a robust, albeit indirect, surrogate biomarker for severe microvascular disorganization. Alternatively, and equally importantly, it must be emphasized that *χ*^2^ is fundamentally a mathematical model-fitting residual rather than a direct biological parameter. Its significant elevation in malignant foci likely reflects a ‘model mismatch’—indicating that the standard Tofts compartmental model fails to adequately capture the highly aberrant and complex kinetics of prostate cancer.

Regarding the relationship with tumor grading, the literature remains divided. Several studies have reported positive associations; for instance, Low et al. [20] investigated 87 patients undergoing pre- and post-radiation DCE-MRI, demonstrating a direct correlation between pre-treatment *K*^trans^ values and Gleason scores, with the highest *K*^trans^ levels noted in GS 9 tumors. Vos et al. [11] concluded that quantitative *K*^trans^ and *K_ep_* maps facilitate discrimination between low-grade and intermediate-to-high grade peripheral zone cancers, though substantial parameter overlap was noted. Similarly, Moradi et al. [21] and Turkbey et al. [22] documented positive correlations between quantitative DCE maps, Gleason grade, and histopathological tumor volume.

Conversely, in a systematic review and meta-analysis comprising 13 studies and 894 patients, Meyer et al. [23] reported only weak correlations between *K*^trans^, *K_ep_*, and Gleason score, with marked parameter overlap between clinically significant (GS ≥ 7) and non-significant (GS < 7) disease. They concluded that current DCE-MRI modeling alone cannot reliably replace histopathological grading. In our study, while pharmacokinetic parameters cleanly separated malignant tissue from normal parenchyma, Kruskal–Wallis testing and Spearman correlation analysis revealed no statistically significant associations between any DCE-MRI metric and Gleason score subgroups (all *p* > 0.05). This reflects the substantial parameter overlap documented by Meyer et al., emphasizing that pharmacokinetic DCE metrics should be interpreted as complementary functional tools rather than standalone substitutes for histopathology. In fact, our ROC analysis for differentiating high-risk (GS ≥ 8) from low-to-intermediate risk (GS 6–7) disease demonstrated that DCE parameters performed poorly, failing to provide incremental predictive value.

Regarding laboratory markers, the utility of serum PSA and PSA density in predicting tumor aggressiveness is well-established. Consistent with extensive prior literature [24,25], our cohort demonstrated that serum PSA, manual PSA density (PSA-D*_m_*), and workstation PSA density (PSA-D*_w_*) were significantly elevated in Group 3 (GS ≥ 8) compared to Groups 1 and 2 (e.g., median PSA: 12.99 ng/mL vs. 6.54 and 6.20 ng/mL, respectively; *p* = 0.004). Furthermore, ROC analysis confirmed that serum PSA (AUC = 0.798) and PSA-D*_m_* (AUC = 0.765) effectively discriminate high-risk disease (GS ≥ 8) from low-to-intermediate risk tumors, significantly outperforming the DCE-MRI parameters for this specific binary classification.

Several limitations of our study warrant mention. First, its retrospective single-center design and relatively small sample size (*n* = 43) limit statistical power and multi-center generalizability. Second, the majority of patients were diagnosed via TRUS-guided cognitive fusion needle biopsy rather than whole-mount radical prostatectomy specimens, introducing potential histological under-grading relative to ultimate surgical pathology. Third, systemic patient factors such as cardiac output, vascular compliance, age, and body weight were not adjusted within the Tofts pharmacokinetic model, which might introduce slight variations in derived arterial input functions. Fourth, there is an inherent selection bias in our study design. As detailed in our patient selection flowchart, our final cohort required a distinct focal target lesion on mpMRI to allow for precise ROI placement and pharmacokinetic modeling. Consequently, we inherently excluded MRI-invisible tumors and low PI-RADS lesions. As a result, our findings are primarily applicable to MRI-visible, higher-suspicion lesions and may not fully reflect the perfusion characteristics of the entire prostate cancer spectrum. Fifth, due to the technical constraints of the post-processing software utilized during the study period (syngo.via), pharmacokinetic parameters were derived from 2D ROIs placed on the maximum cross-sectional area of the lesion rather than through whole-tumor volumetric 3D voxel-by-voxel analysis. We acknowledge that this two-dimensional approach introduces potential sampling bias and may not fully capture the spatial microvascular heterogeneity present throughout the entire tumor volume. Sixth, our subgroup analysis comparing pharmacokinetic parameters across Gleason score groups (GS 6, 7, and ≥8) yielded no statistically significant differences or correlations. This lack of significance is likely attributable to the small overall sample size and the particularly underpowered nature of our intermediate-risk GS 7 subgroup (*n* = 9), which limits the statistical power required to detect subtle microvascular perfusion variations between different tumor grades. Seventh, all imaging acquisitions in this study were performed on a 1.5-T MRI system. Because 1.5-T scanners inherently possess a lower signal-to-noise ratio (SNR) compared to the 3.0-T systems frequently used in contemporary literature, they may require slight compromises in temporal resolution during DCE sequences. Consequently, the absolute pharmacokinetic values and the specific diagnostic cut-off thresholds derived in our study are protocol-specific and cannot be directly generalized or extrapolated to data acquired on 3.0-T MRI platforms. Finally, it is crucial to note that our definition of ‘normal’ background tissue was based entirely on imaging morphology (homogeneously hyperintense peripheral zone on T2WI without restricted diffusion or focal enhancement). Because our study cohort exclusively comprised patients with malignant disease without systematic whole-mount histopathological mapping, we cannot definitively rule out the presence of microscopic disease or diffuse inflammatory changes in these reference ROIs. Therefore, imaging-defined normality does not strictly equate to histopathological normality. Future prospective multi-center trials incorporating standardized software algorithms and larger patient cohorts are recommended to validate these thresholds.

## 5. Conclusions

In this retrospective, single-center study, quantitative and semi-quantitative pharmacokinetic metrics derived from DCE-MRI (*K*^trans^, *K_ep_*, *iAUC*, *V*_e_, *χ*^2^) provided objective functional data that significantly distinguished MRI-visible malignant prostate lesions from normal appearing background parenchyma within our specific patient cohort. However, the derived diagnostic thresholds—most notably the *K*^trans^ cut-off of 0.105 min^−1^—are strictly protocol-dependent, having been acquired exclusively on a 1.5-T scanner and processed via the Siemens syngo.via Tofts model. Consequently, these absolute values cannot be directly generalized across different magnetic field strengths, MR vendors, contrast agents, or post-processing software packages without rigorous cross-platform standardization. Future prospective, multi-center trials evaluating larger cohorts are required to validate these specific thresholds before quantitative DCE-MRI metrics can be reliably incorporated into routine clinical workflows.

## Figures and Tables

**Figure 1:**
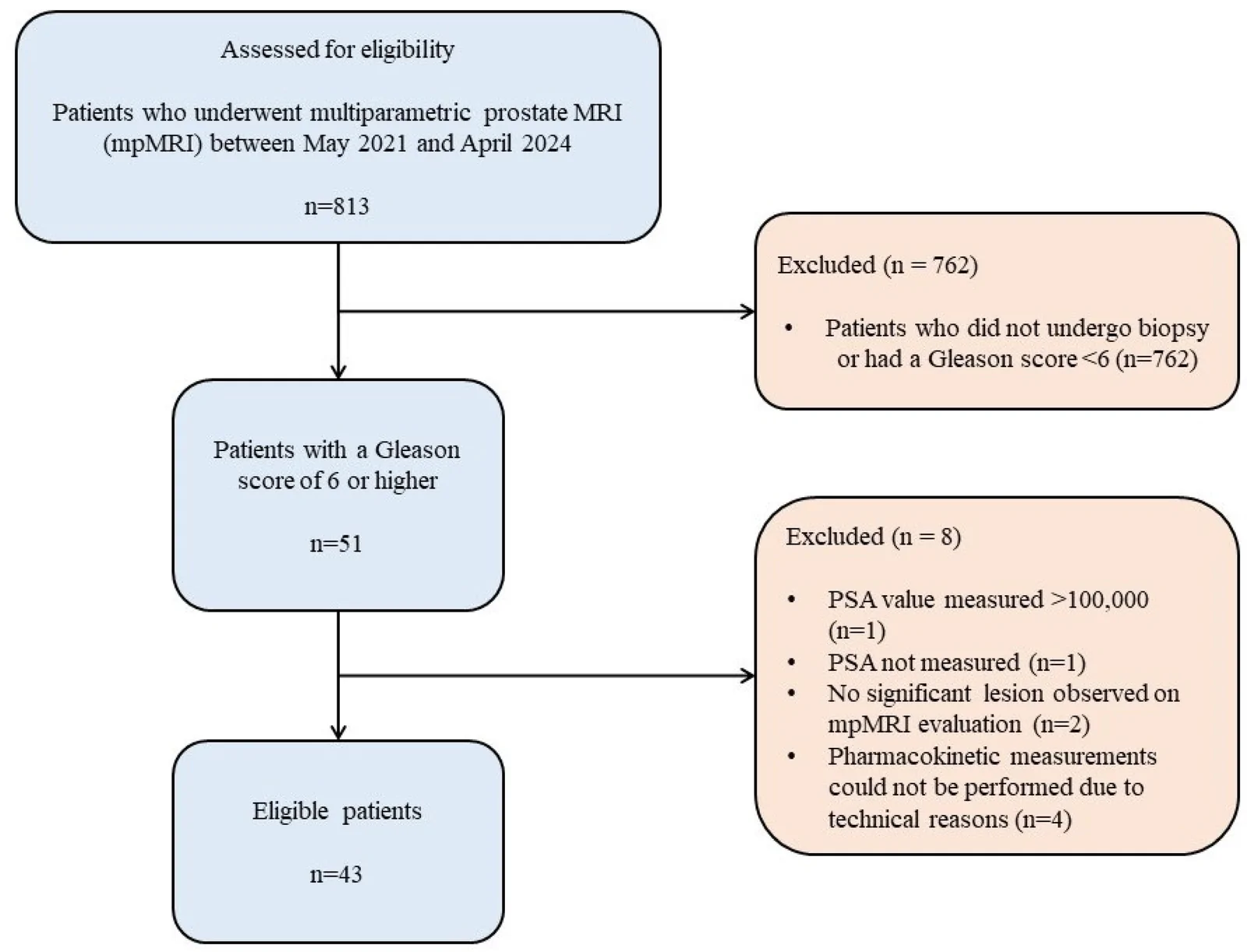
Flowchart of the patient selection process.

**Figure 2:**
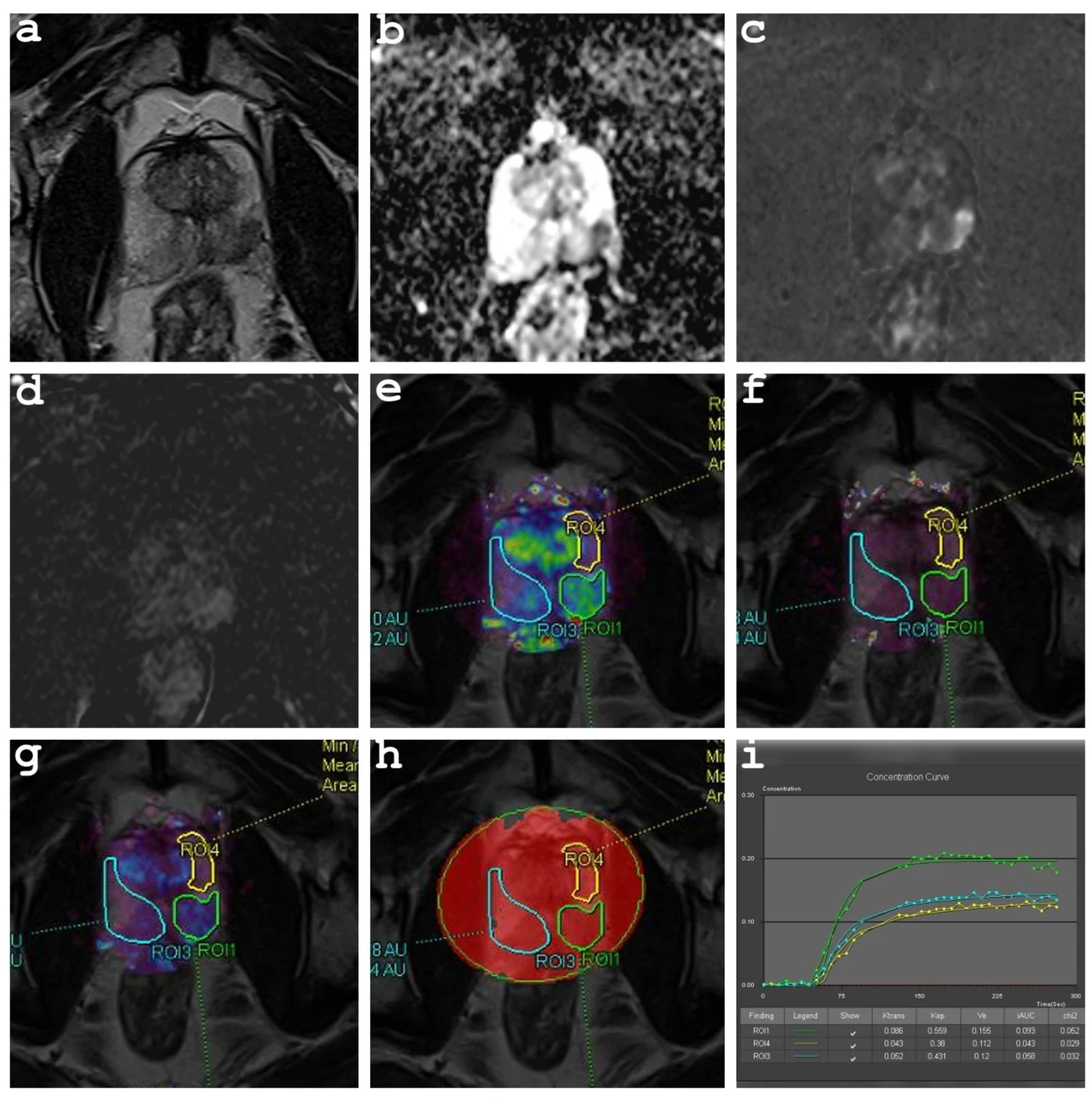
Representative multiparametric MRI (mpMRI) and pharmacokinetic measurement technique for a focal peripheral zone lesion. (**a**) Axial T2-weighted imaging demonstrates a focal hypointense nodular lesion in the left posterior peripheral zone. (**b**) The corresponding apparent diffusion coefficient (ADC) map and (**c**) high b-value (b = 1600) diffusion-weighted image confirm restricted diffusion. (**d**) Dynamic contrast-enhanced (DCE) MRI exhibits early focal enhancement (PI-RADS 4). (**e**–**h**) To extract quantitative metrics, free-hand regions of interest (ROIs) were carefully delineated on the post-processing workstation. A target ROI (green outline) was drawn to strictly encompass the cross-sectional area of the index lesion. For internal comparative analysis, reference ROIs (blue and yellow outlines) were placed over morphologically normal, homogeneous hyperintense peripheral zone tissue within the same axial slice. Parametric color maps generated via the pharmacokinetic model visualize the spatial distribution of (**e**) *K*^trans^, (**f**) *K_ep_*, (**g**) *iAUC* and (**h**) *V_e_*, highlighting distinct localized hyperperfusion within the target lesion compared to the surrounding normal parenchyma. The corresponding time-intensity dynamic concentration curves (**i**) plot the contrast kinetics, displaying a Type 2 (plateau) curve for the target lesion (green ROI) contrasted with Type 1 (persistent linear enhancement) curves for the normal background tissues, thereby facilitating a direct quantitative comparison.

**Table 1:** Multiparametric prostate MRI acquisition parameters.

Sequence	Slice Thickness (mm)	Number of Slices	TE (ms)	TR (ms)	Field of View (cm)	b-Value (s/mm^2^)	Flip Angle (°)	NEX
T2WI Axial TSE	3.0	25	96.0	7400.0	20 × 20	-	144	3
T2WI Coronal TSE	3.0	21	96.0	6040.0	20 × 20	-	148	3
T2WI Sagittal TSE	3.0	21	105.0	6000.0	20 × 20	-	151	3
DWI Axial	3.0	23	103.0	4300.0	22 × 22	50, 400, 800, 1600	90	10
T1WI Axial TSE	3.0	25	11.0	625.0	20 × 20	-	160	1
T1WI VIBE Axial Dynamic	3.0	24	1.79	4.6	22 × 22	-	12	1
Post-contrast T1WI Axial TSE	3.5	50	11.0	655.0	27 × 38	-	160	1

TSE: Turbo Spin Echo, TE: Time of Echo, TR: Time of Repetition, VIBE: Volumetric Interpolated Breath-hold Examination, NEX: Number of Exitations.

**Table 2:** Baseline demographic, clinical, and volumetric parameters of all included patients (*n* = 43).

Variable	Mean ± SD/Median *	Minimum	Maximum
Age (years)	64.21 ± 8.82	47	85
Serum PSA (ng/mL)	6.55 (5.46–10.97) *	3.21	120.20
PV*_m_* (mL)	58.00 (44.95–79.25) *	24.40	138.00
PSA-D*_m_*	0.13 (0.07–0.26) *	0.03	1.64
PV*_w_* (mL)	57.81 (39.05–73.58) *	25.57	162.00
PSA-D*_w_*	0.14 (0.08–0.23) *	0.02	1.54

SD: Standard Deviation; PV*_m_*: Manual Prostate Volume; PSA-D*_m_*: Manual PSA Density; PV*_w_*: Workstation Prostate Volume; PSA-D*_w_*: Workstation PSA Density. * Non-normally distributed variables are presented as Median (Interquartile Range).

**Table 3:** Inter-group comparison of demographic, laboratory, and volumetric variables according to Gleason score categories.

Variable	Group 1 Mean ± SD/Median * (GS = 6, *n* = 20)	Group 2 Mean ± SD/Median * (GS = 7, *n* = 9)	Group 3 Mean ± SD/Median * (GS ≥ 8, *n* = 14)	*p*-Value
Age (years)	63.15 ± 10.25	61.78 ± 3.83	67.29 ± 8.03	0.268
Serum PSA (ng/mL)	6.54 (4.96–9.35) * ^b^	6.20 (3.74–6.30) * ^b^	12.99 (6.78–67.50) * ^a^	0.004
PV*_m_* (mL)	60.50 (44.52–75.43) *	66.00 (47.00–67.00) *	49.35 (45.58–83.25) *	0.950
PSA-D*_m_*	0.11 (0.06–0.20) * ^b^	0.08 (0.05–0.22) * ^b^	0.34 (0.12–1.01) * ^a^	0.019
PV*_w_* (mL)	57.53 (38.45–68.73) *	57.81 (43.14–60.22) *	65.70 (41.16–81.01) *	0.567
PSA-D*_w_*	0.14 (0.07–0.20) * ^b^	0.10 (0.05–0.19) * ^b^	0.31 (0.12–0.95) * ^a^	0.034

SD: Standard Deviation; GS: Gleason Score; PV*_m_*: Manual Prostate Volume; PSA-D*_m_*: Manual PSA Density; PV*_w_*: Workstation Prostate Volume; PSA-D*_w_*: Workstation PSA Density. * Non-normally distributed variables are presented as Median (Interquartile Range). ^a,b^ Superscript letters indicate significant differences between group means based on post-hoc pairwise comparisons (*p* < 0.05).

**Table 4:** Receiver Operating Characteristic (ROC) curve analysis of serum PSA and PSA density parameters for predicting high-grade disease (Group A vs. Group B).

Variable	Area Under the Curve (AUC)	Cut-Off Value	*p*-Value	Sensitivity (%)	Specificity (%)
Serum PSA (ng/mL)	0.798	7.25	0.002	71.4	69.0
PSA-D*_m_*	0.765	0.135	0.005	71.4	62.1
PSA-D*_w_*	0.739	0.145	0.012	64.3	58.6
L-*iAUC*	0.634	0.359	0.162	42.9	82.8
L-*χ*^2^	0.623	0.096	0.199	50.0	79.3
L-*V_e_*	0.567	0.202	0.492	100.0	24.1
L-*K*^trans^ (min^−1^)	0.560	0.129	0.534	85.7	37.9
L-*K_ep_* (min^−1^)	0.516	0.767	0.876	42.9	86.2

PSA-D*_m_*: Manual PSA Density; PSA-D*_w_*: Workstation PSA Density. L: Target lesion measurement; *K*^trans^: Volume transfer coefficient; *K_ep_*: Rate constant; *V_e_*: Extravascular extracellular space volume fraction; *iAUC*: Initial area under curve; *χ*^2^: Goodness-of-fit metric.

**Table 5:** Pharmacokinetic parameter comparison between malignant lesions (L) and normal reference tissue (N) across patient groups.

Variable	All Patients (*n* = 43)	Group 1 (*n* = 20)	Group 2 (*n* = 9)	Group 3 (*n* = 14)	*p*-Value (All)	*p*-Value (G1)	*p*-Value (G2)	*p*-Value (G3)
L-*K*^trans^ (min^−1^)	0.183 ± 0.091	0.189 ± 0.101	0.157 ± 0.074	0.192 ± 0.090	<0.001	<0.001	0.004	0.001
N-*K*^trans^ (min^−1^)	0.056 (0.042–0.101) *	0.052 (0.042–0.101) *	0.086 (0.041–0.094) *	0.058 (0.048–0.099) *				
L-*K_ep_* (min^−1^)	0.549 ± 0.253	0.592 ± 0.243	0.459 ± 0.217	0.545 ± 0.289	<0.001	<0.001	0.020	0.017
N-*K_ep_* (min^−1^)	0.342 ± 0.145	0.359 ± 0.144	0.326 ± 0.117	0.327 ± 0.168				
L-*V_e_*	0.349 (0.237–0.477) *	0.285 (0.188–0.493) *	0.334 (0.246–0.481) *	0.367 (0.271–0.412) *	<0.001	0.002	0.004	0.025
N-*V_e_*	0.202 (0.136–0.346) *	0.162 (0.108–0.376) *	0.202 (0.181–0.225) *	0.240 (0.170–0.334) *				
L-*iAUC*	0.243 ± 0.135	0.228 ± 0.139	0.211 ± 0.123	0.284 ± 0.136	<0.001	<0.001	0.004	0.001
N-*iAUC*	0.087 (0.046–0.142) *	0.072 (0.045–0.136) *	0.126 (0.038–0.167) *	0.095 (0.068–0.165) *				
L-*χ*^2^	0.076 (0.053–0.100) *	0.076 (0.048–0.092) *	0.059 (0.053–0.091) *	0.087 (0.059–0.161) *	<0.001	<0.001	0.004	<0.001
N-*χ*^2^	0.035 (0.024–0.049) *	0.034 (0.024–0.044) *	0.024 (0.022–0.042) *	0.048 (0.030–0.057) *				

* Non-normally distributed variables are presented as Median (Interquartile Range). L: Target lesion measurement; N: Normal reference background measurement; *K*^trans^: Volume transfer coefficient; *K_ep_*: Rate constant; *V_e_*: Extravascular extracellular space volume fraction; *iAUC*: Initial area under curve; *χ*^2^: Goodness-of-fit metric.

**Table 6:** Diagnostic performance and cut-off values of DCE-MRI pharmacokinetic parameters for discriminating prostate cancer from normal tissue.

Parameter	Area Under the Curve (AUC)	Cut-Off Value	*p*-Value	Sensitivity (%)	Specificity (%)
*K*^trans^ (min^−1^)	0.853	0.105	<0.001	81.4	76.7
*χ* ^2^	0.844	0.049	<0.001	79.1	76.7
*iAUC*	0.808	0.136	<0.001	72.1	69.8
*K_ep_* (min^−1^)	0.754	0.405	<0.001	69.8	67.4
*V_e_*	0.706	0.227	<0.001	76.7	65.1

*K*^trans^: Volume transfer coefficient; *χ*^2^: Goodness-of-fit metric; *iAUC*: Initial area under curve; *K_ep_*: Rate constant; *V_e_*: Extravascular extracellular space volume fraction.

**Table 7:** Comparison of quantitative DCE-MRI parameters across Gleason score groups and their correlation with tumor grade.

Pharmacokinetic Parameter	Kruskal–Wallis Test (*p*-Value) *	Spearman’s Correlation (ρ)	Spearman *p*-Value
L-*K*^trans^ (min^−1^)	0.730	0.051	0.747
L-Kep (min^−1^)	0.445	−0.071	0.653
L-*V_e_*	0.755	0.115	0.464
L- *iAUC*	0.350	0.167	0.283
L-χ^2^	0.399	0.146	0.350

* Comparison across GS 6, GS 7, and GS ≥ 8 subgroups. L: Target lesion measurement; *K*^trans^: Volume transfer coefficient; *χ*^2^: Goodness-of-fit metric; *iAUC*: Initial area under curve; *K_ep_*: Rate constant; *V_e_*: Extravascular extracellular space volume fraction.

## Data Availability

The datasets analyzed during the current study are available from the corresponding author upon reasonable academic request, subject to ethical committee privacy restrictions.

## Abbreviations

The following abbreviations are used in this manuscript:

PCa	Prostate Cancer
mpMRI	Multiparametric Magnetic Resonance Imaging
DCE-MRI	Dynamic Contrast-Enhanced Magnetic Resonance Imaging
PI-RADS	Prostate Imaging Reporting and Data System
GS	Gleason Score
PSA	Prostate-Specific Antigen
PSA-D	Prostate-Specific Antigen Density
ROI	Region of Interest
EES	Extravascular Extracellular Space
AUC	Area Under the Curve
TRUS	Transrectal Ultrasound
DRE	Digital Rectal Examination
BPH	Benign Prostatic Hyperplasia
